# New evolving strategies revealed by transcriptomic analysis of a *fur* ^*−*^ mutant of the cyanotrophic bacterium *Pseudomonas pseudoalcaligenes* CECT 5344

**DOI:** 10.1111/1751-7915.13408

**Published:** 2019-04-21

**Authors:** Gracia Becerra, María Isabel Igeño, Faustino Merchán, Rubén Sánchez‐Clemente, Rafael Blasco

**Affiliations:** ^1^ Departamento de Bioquímica y Biología Molecular y Genética Facultad de Veterinaria Universidad de Extremadura Caceres Spain; ^2^ Meat and Meat Products Research Institute (IProCar) BioMic Research Group Universidad de Extremadura Caceres Spain

## Abstract

The transcriptomic analysis (RNA‐seq) of a *fur* mutant of *P. pseudoalcaligenes* CECT 5344 has revealed that Fur regulates the expression of more than 100 genes in this bacterial strain, most of them negatively. The highest upregulated genes in response to *fur* deletion, with respect to the wild type, both cultivated in LB medium, corresponded to genes implicated in iron uptake. They include both TonB‐dependent siderophore transporters for the active transport across the outer membrane, and ABC‐type and MSF‐type transporters for the active transport across the cytoplasmic membrane. Therefore, the main response of this bacterium to iron limitation is expressing genes necessary for metabolism of Fe siderophores produced by other microorganisms (xenosiderophores). The number of genes whose expression decreased in the *fur*− mutant, as well as its normalized expression (fold change), was lower. Among them, it is remarkable the presence of one of the two *cas* operons of the two CRISP/Cas clusters was detected in the genome of this bacterium. The transcriptome was validated by qPCR, including the decrease in the expression of *cas* genes (*cse1*). The expression of *cse1* was also decreased by limiting the amount of iron, carbon or nitrogen in the medium, or by adding menadione, a compound that causes oxidative stress. The higher decrease in *cse1* expression was triggered by the addition of cyanide in minimal medium. These results suggest that this bacterium responds to stress conditions, and especially to cyanide, taking a reasonable risk with respect to both the uptake of (TonB‐dependent receptors gates) and the tolerance to (reduced immunity) foreign nucleic acids. In conjunction, this can be considered a yet unknown molecular mechanism forcing bacterial evolution.

## Graphical abstract

Cyanide triggers iron deprivation that, in a process mediated by the master regulator protein Fur, induce the expression of TonB‐dependent receptors gates that may serve for the entrance of foreign nucleic acids. At the same time, in *P. pseudoalcaligenes* CECT 5344, cyanide repress the expression of a CRISP‐Cas system. The presence of Fur boxes upstream the TonB dependent receptors and the Cas proteins suggest a direct regulation of these systems by holo‐Fur, repressing the expression of the former but promoting the expression of the CRISPR‐Cas system. Moreover, the presence of cyanide was more effective than iron starvation in repressing the expression of the CRISPR‐Cas system. Therefore, cyanide seems to drive bacterial evolution, at least in this strain.

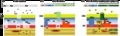

## Introduction

Iron is an essential metal for most biological systems because it is the cofactor of many enzymes participating in central metabolic processes. For example, cytochromes and iron–sulfur proteins are essential components of the respiratory chains. In these enzymes, iron acts as a redox centre, but in many other enzymes such as fumarase or aconitase, the redox state of iron remains constant. Although the concentration of iron in the earth crust is higher than its abundance in living beings, its bioavailability is scarce; thus, organisms have evolved active mechanism for iron acquisition. It is widely accepted that the low concentration of soluble iron limits surface ocean productivity, probably due to limitation of N_2_ fixation, even though iron participates as cofactor in many other reactions of central biogeochemical cycles (Morel and Price, 2003). The archetypical bacterial response to iron limitation is the synthesis and release of small organic molecules, called siderophores, which have high affinity for Fe(III). Iron starvation also induces the synthesis of proteins needed for the active transport of the chelated iron. Therefore, the global response to iron starvation needs the simultaneous function of, at least, regulatory, biosynthetic and transport genes. All these operons usually cluster together in bacterial chromosomes, although selective pressure may change this general rule (Bruns *et al*., 2017). In any case, iron deprivation activates mechanisms leading to iron accumulation inside the cells, due to its essentiality. On the other side, iron(II) catalyses the oxidation of organic matter by O_2_ (Fenton reaction). In the intracellular milieu, ROS production damages macromolecules and lipids (Touati, 2000; da Silva Neto *et al*., 2009). Therefore, an excess of iron is problematic because it promotes the formation of reactive oxygen species (ROS) through the Fenton reaction. For the above‐mentioned reasons, the maintenance of the appropriate concentration of iron is critical. This process involves the balance of iron uptake, intracellular demand and iron storage. The main regulatory protein controlling bacterial iron homeostasis is Fur (ferric uptake regulator). Fur, which reversibly binds Fe^2+^, when loaded with iron (*holo*‐Fur) represses the transcription of several genes implicated in iron uptake. If we assume that the apo‐protein does not have regulatory properties, this simple mechanism may explain how to adjust iron acquisition to the intracellular iron concentration in bacteria. However, reality is not that simple, and it has been shown that *holo*‐Fur may act as a positive regulatory element (Foster and Hall, 1992; Yu *et al*., 2016). In the simplest case, the repression of a gene acting as repressor may explain this positive regulation. The role of *holo*‐Fur as repressor of positive regulator has been described in some reviews (Troxell and Hassan, 2013). When Fur regulates the expression of regulatory elements, the regulatory network of Fur becomes considerably expanded (Yu *et al*., 2016). *Holo*‐Fur may also behave as a direct positive regulator of transcription by displacing a silencer (Nandal *et al*., 2010). Finally, *apo*‐Fur may also interact directly with DNA regulating the expression of target genes (Ernst *et al*., 2005; Carpenter *et al*., 2009). Fur, as a master regulator, recognizes many target sites referred to as the Fur boxes. Originally, the Fur box refers to specific upstream DNA sequences interacting with iron‐loaded Fur (*holo*‐Fur), repressing the expression of its target genes. Nevertheless, the structural characterization of the interaction of *holo*‐ and *apo*‐Fur with DNA reveals that the interaction may have two components, the base readout itself and a readout‐shaped component (Deng *et al*., 2015). Various models of Fur‐DNA binding sites have been proposed, including a single 9‐1‐9 inverted repeat (Chen *et al*., 2007), a head‐to‐head‐to‐tail 6‐mer repeat (Escolar *et al*., 1998) and a minimal 7‐1‐7 repeat (Butcher *et al*., 2011). Therefore, the sequence recognized by Fur seems to be a highly degenerate AT‐rich region, but the molecular mechanisms and structural basis of Fur‐DNA binding are still unclear, and its study remains under intense investigation (Deng *et al*., 2015; Sarvan *et al*., 2018).

Iron metabolism is especially important in bacterial pathogens because iron acquisition from the host is key to survival, and hence, iron starvation is a virulence signal in several pathogens. The regulatory network of Fur in some bacterial pathogens has been studied by using transcriptomic analysis, either DNA microarray (Grifantini *et al*., 2003) or most recently RNA‐seq (Yu *et al*., 2016).

The CECT 5344 bacterial strain of *Pseudomonas pseudoalcaligenes* has been isolated by enrichment cultivation at alkaline pH, thus minimizing cyanhydric acid volatilization, and using cyanide as the sole nitrogen source (Luque‐Almagro *et al*., 2005). The concentration of free iron in the culture media was supposed to be very low. Later on, we have provided experimental evidence that cyanide induces iron deprivation in *P. pseudoalcaligenes* CECT5344, as evidenced by the induction of the expression of *fiu*A (from ferrichrome receptor), as well as the small non‐coding RNA *prrF* (Becerra *et al*., 2014). The *fur*− mutant showed a slow‐growing phenotype, especially in minimal culture medium, and also an increased sensitivity to cyanide in LB medium (Becerra *et al*., 2014). The relationship between iron and cyanide metabolism is also evidenced by the fact that iron has a stimulatory effect on cyanogenesis (Askeland and Morrison, 1983).

Although there are genes regulated by Fur but not repressed in the presence of iron (Ho and Ellermeier, 2015), in general the inactivation of Fur mimics the complete absence of iron in the culture media. In this sense, and taking into account that cyanide generates a signal equivalent to iron starvation, the main objective of this manuscript is to discover the response of *P. pseudoalcaligenes* CECT 5344 to iron limitation in order to distinguish the genes regulated by Fur from those having a specific response to cyanide.

## Results

**Figure 1:**
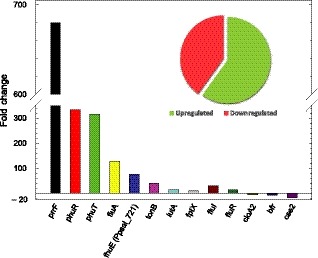
Global effect of the deletion of the *fur* gene in the transcriptome of *Pseudomonas pseudoalcaligenes* CECT5344. The bars indicate the fold change of the some of the representative genes whose expression changed most, either positively (positive *y*‐axis) or negatively (negative *y*‐axis), as a consequence of the mutation of the *fur* gene. The inserted figure (pie diagram) summarizes the percentage of genes whose expression increased (green) or decreased (red) due to the elimination of the *fur* gene.

A transcriptomic analysis of a *fur*‐minus mutant, in comparison with the wild‐type strain, both cultured in LB medium, has been carried out as indicated in the experimental procedures. The results covered the full genome, and the data were analysed according to accuracy (*P*‐value < 0.05) and fold change (Table S1). We found that the expression level of 216 genes with a *P*_val_ < 0.05 varied significantly (fold change of 3–4, Table S2). Since many genes are organized in operons in bacteria, the relative concentration of these mRNAs tends to appear relatively grouped due to their similar fold change, but some operons were shuffled. In order to avoid that, genes were later manually grouped in operons based on three principles: first, the detection of experimental reads in the intergenic sequences of the operon; second, the prediction of the existence of the operon based on informatics programs (softberry); and third, the existence of bibliographic information. The genes with negative fold changes were ordered and grouped at the end of the table following the same rules. The application of these rules allows us to end up with a reduced table (Table S2). From Table S2, it becomes evident that the genes with the highest fold change were those upregulated in the *fur*− mutant (Fig. 1). Moreover, the number of genes upregulated was higher (60%) than the genes downregulated in the *fur*− mutant (40%) (Fig. 1 – inset).

**Figure 2:**
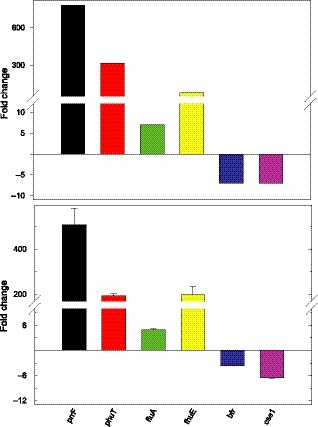
Validation of the transcriptomic data. Comparison of the fold change in the expression of the indicated genes, due to the mutation of the *fur* gene, measured by qPCR in comparison with the transcriptomic data.

The validation of the transcriptomic data was performed by analysing the expression of some representative genes by qPCR (Fig. 2). The endogenous gene employed as reference in the qPCR experiments was the 16S RNA gene, although similar results were obtained by using *rpoD* (not shown). In any case, it is evident that the qPCR analysis validates the transcriptomic data.

**Figure 3:**
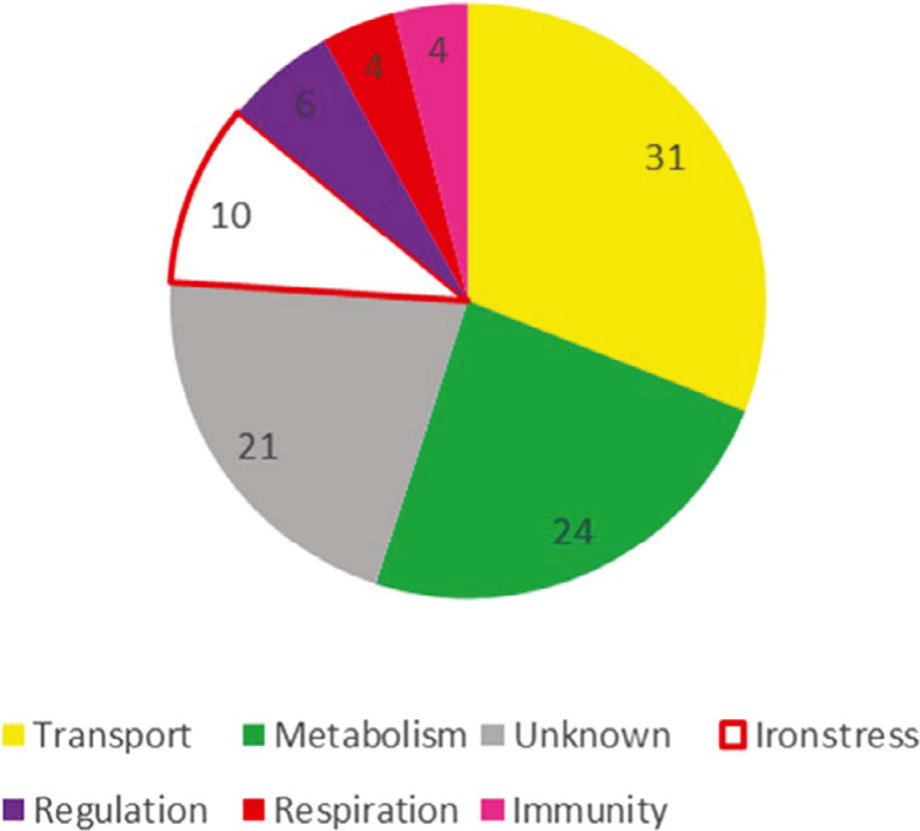
Functionality of the genes regulated by Fur. The genes whose expression significantly changed were grouped according to the indicated functions and the percentage of genes belonging to each function calculated from Table S2.

In a first analysis, the genes whose expression becomes significantly modified by the absence of Fur can be classified into seven general categories, namely iron transport (30.9%), metabolism (24%), stress response and iron metabolism (9.7%), regulation (6.2%), respiration (4.6%), bacterial immunity (CRISPR/Cas) (4%) and unknown functions (20.6%) (Fig. 3).

**Figure 4:**
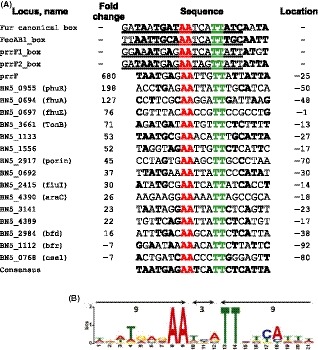
The consensus Fur box.A. Alignment of the sequences of the canonical Fur box, the Fur‐*feoAB1* box, and the *prrF1* and *prrrF2* boxes (underlined) with putative Fur boxes of 16 genes regulated by Fur in *P. pseudoalcaligenes*. The consensus Fur box centres in a – AANNNTT – motif that is conserved in all the analysed regions. Bold nucleotides reveal complementarity in the predicted inverted repeat structure. The location of the region refers to the first nucleotide of the consensus sequence with respect to +1 site.B. Sequence logos representing the motifs generated by MEME.

The next goal was to look for the putative Fur boxes upstream of the regulated genes. The most widely accepted Fur box is a 19 base long, AT‐rich, whose consensus sequence is 5′‐GATAATGAT**AA**TCA**TT**ATC‐3′ (Wilderman *et al*., 2004; Deng *et al*., 2015), that can be seen as a perfect 9‐1‐9 inverted repeat. The minimal requirement parameters to constitute a Fur box should identify a match of, at least, 14 bases of the consensus and a stem of 7–12 complementary bases with a loop of 5–9 bases (Wilderman *et al*., 2004). Following these rules, the only putative Fur box found in the 200 bases upstream Fur transcriptome was detected in the promoter region of *prrF*. In a previous study, we have demonstrated that the expression of *prrF* in the *fur*− mutant of *P. pseudoalcaligenes* increases almost 700‐fold (Becerra *et al*., 2014). Taking this sequence as reference, as well as the conserved *prrf1* and *prrf2* boxes of *Pseudomonas aeruginosa* (Wilderman *et al*., 2004), plus the consensus Fe(II)‐Fur‐binding sequence and the Fur box in the *feoAB1* operator of *P. aeruginosa* (Deng *et al*., 2015), it is possible to infer a new interpretation for the Fur box (Fig. 4). This sequence is 21 base long organized as a 9‐3‐9 inverted repeats centred at the **AA**NNN**TT** motif of the canonical Fur box (positions 10‐16, 5′→3′) (Fig. 4A). We found this consensus sequence in 16 upstream intergenic sequences of Fur‐regulated genes, 14 negatively and 2 positively (Fig. 4A). The two central inverted repeats around the triplet (positions 8, 9 and 13,14) are fully conserved, not only in *Pseudomonas* and *E. coli*, but also in the Fur box of *V. cholerae*,* C. jejuni* and *B. subtilis* (Deng *et al*., 2015). This consensus is very similar to the 7‐1‐7 inverted repeat Fur box of *Pseudomonas syringae* deduced by ChIP‐seq (Butcher *et al*., 2011). Moreover, the central motif of the revised Fur box seems to be in the boundary between nucleic acids bound by the two monomers of Fur in the *feoAB1* promoter of *Pseudomonas aeruginosa* (Deng *et al*., 2015). The proposed consensus sequence is a perfect 9‐3‐9 inverted repeat (Fig. 4). In contrast to the fully conserved – **AA**NNN**TT** – central motif, the remaining 14 bases seem to be degenerated among the different Fur boxes. Nevertheless, they show a variable complementarity (Fig. 4A) that can be related to the stability of the Fur‐DNA complex. Therefore, both the strength of the interaction and the distance of the promoter, among other factors, may be related to the variable response to iron concentration in the expression of different Fur‐regulated genes. In addition to the upstream region of *prrF*, a putative Fur box was detected in the intergenic region of *phuR* and *phuS*, the protein‐coding gene that showed highest fold change (Fig. 4). Phu, from *Pseudomonas* haem uptake (Ochsner *et al*., 2000), includes PhuR that is the TonB‐dependent outer membrane transporter. The expression level of BN5_0956 gene (*phuS*) was 333‐fold higher in the mutant than in the WT. The genes of this locus, from BN5_0594 to BN5_0961, were all upregulated in the *fur*− mutant (Table S2). Although consecutive in the genome, these genes seem to form two divergently transcribed transcription units, one containing two genes (BN5_0955‐BN5_0954) and the other one‐six (BN5_0956 to BN5_0961). The homologous *phu* (*Pseudomonas* Haem Uptake) operon in *P. aeruginosa* also includes *phuR*, a TonB‐dependent haem receptor, divergently transcribed from *phuSTUVW*. PhuS is probably necessary for the metabolism of haem, whereas BN5_0961 is a hypothetical protein of unknown function. The rest of the operon codes the ABC‐type transporter needed for the transport of haem through the inner membrane (Ochsner *et al*., 2000). In total, eleven genes belonging to the ABC‐type transporter family were overexpressed in the *fur*− mutant, whereas six were repressed (Table S2). In *P. pseudoalcaligenes* BN5_0954, a putative protein containing a Rieske centre seems to be co‐regulated with *phuR*. *PrrF* is located in the intergenic region downstream BN5_0961, although it is not part of this operon (*phuSTUVW*) since it is expressed in the opposite direction (not shown). There is a single copy of *prrF* in *P. pseudoalcaligenes* CECT 5344 genome, but other sequenced Pseudomonads encode two *prrF* RNAs, either at distal genomic loci or in tandem, as in *P. aeruginosa* strains (Oglesby‐Sherrouse and Vasil, 2010). *PrrF* is homologous to the small regulatory RNA RyhB in *E. coli* that mediates some of the positive regulation exerted by Fur (Massé and Gottesman, 2002). In these cases, Fur does not affect directly the expression of the target gene, but its post‐transcriptional processing. Therefore, these genes are not expected to appear in the *fur*− transcriptome.

The expression level of *fhuA* was 127‐fold higher in the mutant than in the WT. (Table S2). FhuA from *Escherichia coli* is a TonB‐dependent protein that transports the ferric siderophore ferrichrome, and it is the receptor for bacteriophages T5, phi 80 and T1, and for colicin M (Koebnik and Braun, 1993; Bonhivers *et al*., 1996). Ferrichrome is a xenosiderophore that is not synthesized by bacteria but rather by fungi. The downstream sequences, BN5_0695‐6, seem to form an operon with *fhuA*, although expressed at a lower extent. BN5_0695 is a putative iron‐depending hydrolase, whereas BN5_0696 is a putative isomerase. Upstream in this locus, BN5_0692, a putative flavodoxin, could be the first gene of the operon although the *P*‐val of the following gene, BN5_0693, a putative decarboxylase, is not representative (Table S2). Induction of flavodoxin (a non‐iron protein) under iron limitation has been shown in a variety of microorganisms where it may substitute the iron–sulfur protein ferredoxin (Erdner and Anderson, 1999). The presence of a putative Fur box upstream this gene (Fig. 4) suggests a direct regulation of the expression of this operon by *holo*‐Fur.

BN5_0697 (*fhuE*) is also a TonB‐dependent putative siderophore transporters whose expression is significantly increased (76‐fold) in the *fur*− mutant (Table S2). In *E. coli*, the homologous gene codes an outer membrane receptor protein that is induced under iron limitation (Hantke, 1983) and is required for the uptake of iron(III) via coprogen (an iron chelator produced by *Neurospora crassa*), ferrioxamine B and rhodotorulic acid (Sauer *et al*., 1990). In addition to *fhuE*, the genes *fhuCDB*,* tonB* and *exbB* were necessary for iron coprogen uptake (Hantke, 1983). *fhuCB* were also upregulated in the *fur*− mutant of *P. pseudoalcaligenes* CECT 5344 (BN5_0756 and BN5_0757), although they are not in the same operon as in *E. coli* (Hantke, 1983). FhuB, the periplasmic binding protein (PBP), in conjunction with the membrane‐anchored FhuCD, are supposed to be involved in the transport of hydroxamate‐type siderophores (Andrews *et al*., 2003). A Fur box was detected in the upstream regions of *fiuA* and *fiuE* (Fig. 4).

BN5_4388 (*ampG*) codes a putative membrane protein with 12 transmembrane helix 33% identical to PA_4218 from *P. aeruginosa* PAO. This ferripyochelin transporter, called FptX, in conjunction with RhtX (rhizobactin 1021 transporter) from *Sinorhizobium meliloti* belongs to a new family of MSF‐type siderophore transporters constituted by a single protein functionally equivalent to the ABC‐type transporters (Cuiv *et al*., 2004). The genes of the major facilitator superfamily (MFS) are also active transporters that use the chemiosmotic ion gradients of the inner membrane instead of ATP (Pao *et al*., 1998). The expression of 15 genes belonging to the MSF family and four to the RDN family increased in the *fur*− mutant (Table S2). Downstream BN5_4388 (MSF), and constituting an operon, is located BN5_4387 (*fepA*), another putative TonB‐dependent receptor. Nevertheless, the operon seems to begin in BN5_4389, a gene of unknown function. This operon is transcribed in the opposite direction to BN5_4390, a putative regulator of the AraC family, whose expression increases 26‐fold in the *fur*− mutant and shows a putative Fur box (Fig. 4). This gene is homologous to *pchR* from *P. aeruginosa*, a transcriptional regulator that activates the synthesis of the ferripyochelin receptor protein. This gene has also been shown to be under the control of Fur in *P. aeruginosa* (Heinrichs and Poole, 1993). In *vibrio vulnificus,* a ferrioxamine B receptor has been described to be induced via AraC under iron‐limiting conditions (Tanabe *et al*., 2005).

**Figure 5:**
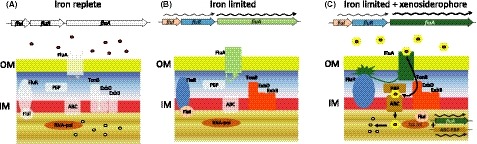
Regulatory genes regulated by Fur, the ECF sigma factor *fiuI*.A. Under iron‐replete conditions, Fur represses the expression of the TonB‐dependent transporter *fiuA*, as well as the expression of the sigma/anti‐sigma couple *fiuI*‐*fiuR*.B. Both operons were transcribed under iron limitation.C. The cognate xenosiderophore in the extracellular media (yellow hexagon) activates FiuA. The interaction of FiuA with FiuR releases FiuI to promote the expression of genes necessary for the transport of this concrete xenosiderophore, including an extra‐expression of *fiuA*. The mechanism schematized in C is speculative and based on bibliographic data, since only the overexpression of *fiuA* and *fiuIR* was determined in the *fur*− mutant in the present study (Table S2).

Among the TonB‐dependent receptors, BN5_2417, whose expression was seven times higher in the Fur mutant than in the wt strain, is homologous (58 identical at the amino acid level) to PA0470 (FiuA), and it shares the same synteny with *fiuI* and *fiuR* (Fig. 5). *fiuA* gene codes a TonB‐dependent receptor of the fungal (xeno)siderophore ferrichrome (Llamas *et al*., 2006). The absence of Fur protein induces (de‐represses) the expression of *fiuA* and *fiuIR* (Table S2), as schematized in Fig. 5. Consistent with this scheme is the presence of putative Fur boxes upstream *fiuA* and *fiuR* (Fig. 4). FiuI is an alternative sigma factor of the extracytoplasmic function (ECF) family. Therefore, this TonB‐dependent receptor may function as a pore or as a sensor for ferrosiderophores (Noinaj *et al*., 2010). In addition to this regulatory circuit and *araC* (BN5_4390), 11 putative regulatory proteins were upregulated in the *fur*− mutant (Table S2). They include response regulator of the two‐component signal transduction pathway type (BN5_2773‐BN5_2774, BN5_1556, BN5_1557, BN5_0759) as well as other putative DNA binding proteins belonging to different subfamilies (BN5_4180, BN5_2140, BN5_1161, BN5_4493).

The relative concentration of the mRNA coding the TonB system itself [BN5_3557 (*tonB*), BN5_3558 (*exbD*) and BN5_3559 (*exbB*)] was around 40 times higher in the *fur*− mutant. BN5_3656, BN5_3660 and BN5_3661 seem to form an operon with the TonB system (Table S2), but its function is unknown. There is a putative Fur box upstream BN5_3661 (Fig. 4). The *N*‐terminal domain of the different TonB‐dependent receptors (located in the outer membrane) interacts with TonB, a protein anchored in the inner membrane. TonB mediates the transport of the ferrisiderophore complex across the outer membrane in conjunction with ExbB and ExbD using the electrochemical potential (Andrews *et al*., 2003; Postle and Kadner, 2003). The upregulation of BN5_1050, BN5_1049, BN5_1048 and BN5_1047 was still considerable. There seems to be fragments of PSPTO3574 and PA0151, a TonB‐dependent siderophore transporter. In total, 17 TonB‐dependent receptors and porins became upregulated in the *fur*− mutant (Table S2).

The expression of BN5_3002 increased 14 times in the mutant. It is 30% identical to PA0740 (*sdsA1*), an alkylsulfatase from *Pseudomonas aeruginosa*. The group of proteins grouped as involved in metabolism is very heterogeneous. Most of them have a predicted enzymatic activity, but it is difficult to assign them to a concrete biological process. This group contains 42 genes, 24 of them overexpressed in the *fur*− mutant (Table S2). The group of genes coding putative proteins of unknown function comprises 36 members, 10 of them overexpressed in the *fur*− mutant. One of them, BN5_1133, shows a Fur box in its upstream region.

There are eight genes, belonging to two operons coding terminal oxidases of the respiratory chain whose expression was significantly lower in the *fur*− mutant (Table S2). BN5_2522, BN5_2523 and BN5_2524 constitute one of the three *cio* operons present in the genome of *P. pseudoalcaligenes* CECT 5344, namely *cio2* (Luque‐Almagro *et al*., 2013). The expression of *cio2* was around 5 times lower in the *fur*− mutant than in the wild type, similar to the decrease in the second *cbb*_*3*_‐type operon found in the genome of this bacterium (Luque‐Almagro *et al*., 2013). No Fur boxes were detected in this case. In *Helicobacter pylori*, apo‐Fur positively regulates the expression of sodB (Ernst *et al*., 2005). The same mechanism may account for the regulation of the expression of *sodB* (BN5_1139) in *P. pseudoalcaligenes*, since its expression diminished sevenfold in the *fur*− mutant, but no Fur boxes were found in its upstream region. The Fur box was also absent in the promoter region of the downregulated genes BN5_3929, BN5_2441, BN5_2521 and BN5_2462, as well as the upregulated genes BN5_0248, BN5_1161, BN5_1164, BN5_0754 and BN5_0755 (Table S2).

**Figure 6:**
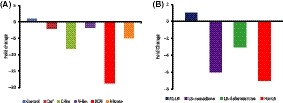
Expression of the *cse1* gene of *P. pseudoalcaligenes* CECT 5344.A. Wild‐type (LB R1) and *fur*− (Fur LB) cultures of *P. pseudoalcaligenes* CECT 5344 were grown in LB medium until reaching the exponential growth phase (OD_600_ ≈ 0.5). At that phase, the mRNA was extracted as indicated in Experimental Procedures. Two additional flasks containing the wt strain cultured in parallel up to the same optical density were treated with either menadione (200 μM) (LB – menadione) or deferoxamine (50 μM) (LB – deferoxamine). One hour after treatment, the relative expression of *cse1* gene was also determined.B. *P. pseudoalcaligenes* CECT 5344 cells were grown in minimal medium with 40 mM acetate and 5 mM ammonium as respective *C*‐ and *N*‐sources. Cells were harvested by centrifugation at the mid‐exponential growth phase (OD600 ≈ 0.4). The relative concentration of the *cse1* mRNA was measured with respect to the 16S rRNA gene transcript (control). The effect of the presence of cyanide or deferoxamine was estimated by measuring the relative concentration of *cse1* mRNA after 1 h of treatment of the culture with 2 mM of cyanide (KCN) or 200 μM of menadione (Def). Carbon limitation and nitrogen limitation were emulated by using 5 mM acetate (C‐lim) and 2 mM ammonium (N‐lim) respectively. In both cases, cells were collected at the stationary phase. Nitrate 5 mM was also used instead of the same concentration of ammonium (control).

The expression of BN5_1112, which codes bacterioferritin (Bfr), an iron storage protein, decreased seven times upon *fur* mutation. In contrast, the expression of BN5_2984, the bacterioferritin‐associated ferredoxin (Bfd), increased 16 times in the Fur mutant. Although these genes are not consecutive in the genome of *P. pseudoalcaligenes* CECT 5344, we propose to maintain the nomenclature accepted for the homologous genes in *E. coli* and *Pseudomonas* because they also show the same pattern of regulation in the context of iron metabolism. The presence of a Fur box in the upstream region of *bfd* is consistent with the original scheme of *holo*‐Fur as transcriptional repressor. Nevertheless, *holo*‐Fur has been demonstrated to induce the expression of *bfr* in *E. coli* by reversal of H‐NS silencing protein at a distal position (Nandal *et al*., 2010). The presence of a putative Fur box 92 bases upstream the + 1 site of BN5_1112 (*bfr*) (Fig. 4) agrees with this model of regulation. The presence a Fur box at a distal position upstream BN5_0768 (*cse1*) (Fig. 4) is also in concordance with a direct interaction of *holo*‐Fur enhancing its expression. This operon (BN5_0768‐BN5_0774) constitutes the CRISPR (Clustered Regulatory Interspaced Short Palindromic Repeats)‐associated proteins (CAS). CRISPR sequences contain short repeat sequence separated by variable sequences derived from invaders such as viruses and conjugative plasmids (Mojica *et al*., 2005; Hale *et al*., 2009). In the complete genome of *P. pseudoalcaligenes* CECT 5344 (Wibberg *et al*., 2014), there are two CRISPR/Cas systems. The CRISPR/Cas can be classified into three major groups attending to the sequence of the Cas proteins as well as to the repetitive sequence (Makarova *et al*., 2011). The two CRISPR/Cas found in *P. pseudoalcaligenes* CECT 5344 belong to the type I, although only the expression of the Cas genes mentioned above, which belong the subtype E (*E. coli*), was decreased in the *fur*− mutant (Table S2). The expression of the other CRISPR/Cas cluster, which spans from BN5_4286 to BM5_4295, and belongs to the subtype F (*Y. pestis*), does not change in the *fur*− mutant. BN5_0768 (*cse1*) was one of the genes used for the validation of the transcriptome (Fig. 2). The decrease in the expression observed in the transcriptome coincided with that measured by qPCR. Moreover, the expression of *cse1* was also decreased by the addition of menadione and by the iron deficiency caused by the addition of deferoxamine (Fig. 6A). In order to check whether the downregulation of Cas is specific to iron limitation, the qPCR experiments were carried out in minimal media containing limiting amounts of carbon, nitrogen or iron (Fig. 6B). The response to iron limitation in minimal medium, triggered by the addition of deferoxamine, was lower than that observed in LB medium, but qualitatively equivalent (Fig. 6). Both carbon and nitrogen limitation also negatively affected the expression of *cse1*, but it was the addition of cyanide that most diminished the expression of this gene (Fig. 6B).

## Discussion

It is widely accepted that Fur protein responds to and senses intracellular Fe(II) concentration with relatively high selectivity, being the master regulator for iron homeostasis in bacteria (Chandrangsu *et al*., 2017). The term *fur* for ‘iron uptake regulation’ was suggested for a new class of mutant that constitutively expresses some high‐affinity iron uptake systems in *Salmonella typhimurium* (Ernst *et al*., 1978). If Fur senses the intracellular concentration of iron, the absence of a functional Fur protein should be equivalent to the complete absence of iron. In other words, a *fur*− mutant is to iron what a diabetic is to glucose. This simple reasoning explains the phenotype of the original *S. typhimurium* mutant (Ernst *et al*., 1978). The mutant constitutively overexpresses high‐affinity iron uptake systems, even in Fe‐loaded media. In view of this, it can be discussed the fact that the main response observed in the *fur*− mutant of *P. pseudoalcaligenes* CECT 5344 was the upregulation of genes involved in iron uptake (Figs 1 and 3 and Table S2). Nevertheless, the genes negatively regulated in the *fur*− mutant could also be of crucial importance for iron metabolism, as we will discuss later. Moreover, it cannot be fully discarded that Fur may have iron‐independent regulatory functions (Yu *et al*., 2016). The adaptation to iron‐restricted conditions usually proceeds in two complementary ways, first by upregulating the expression of ‘genes for iron acquisition’, and second by using alternative metabolic pathways, or isoenzymes, designed to economize iron. Archetypical examples of these processes are the production of siderophores and the repression of the synthesis of iron‐rich proteins respectively. The main function of siderophores is acting as iron‐shuttle, although recent studies have shown that they may have additional functions (Johnstone and Nolan, 2015). The term ‘genes for iron acquisition’ involves the genes both encoding the synthesis of the siderophore itself and active transport of the siderophore. Once again, this transport implies crossing two membranes in Gram‐negative bacteria. The transport across the outer membrane proceeds through the TonB‐dependent receptors. In the classical scheme, the transport across the inner membrane needs a periplasmic binding protein (PBP) and an inner membrane ATP‐binding cassette (ABC) transporter. Nevertheless, a transporter of the MSF family may well substitute the function of the ABC transporter. The *phu* locus (BN5_0954‐0961) is an example of co‐regulation of a TonB‐dependent outer membrane transporter (BN5_0955) with its corresponding ABC‐type transporter (BN5_0958‐60), whereas in the *fepA* locus (BN5_4387‐90), the TonB‐dependent porin (BN5_4387) and a MSF‐type transporter (BN5_4388) seem to be part of the same operon (Table S2). In both cases, Fur boxes were observed in the upstream intergenic regions of the regulated genes (Fig. 4). Bacteria usually have much more TonB‐dependent receptors for siderophores than the number of siderophores synthesized by the bacterium. For example, *P. aeruginosa* has the capacity to synthetize pyoverdine and pyochelin, but 32 additional genes encoding putative TonB‐dependent receptors have been detected in its genome (Llamas *et al*., 2006). In *P. pseudoalcaligenes*, the situation is more radical, because this bacterium does not synthesize any siderophores (Becerra *et al*., 2014). The resulting phenotype resembles a kind of piracy for siderophores, but it may have an ecological benefit in the local microbial community. *P. pseudoalcaligenes* CECT 5344 does not assimilate strong acid dissociable (SAD) cyanide complexes (Luque‐Almagro *et al*., 2005), probably because it does not produce siderophores (Becerra *et al*., 2014). Therefore, the effective biodegradation of cyanide‐containing wastes that usually contain SAD cyanide probably needs the use of mixed cultures of *P. pseudoalcaligenes* CECT 5344 in conjunction with siderophore generating strains. This hypothesis is now under experimentation. Although the expression of TonB‐dependent siderophore receptors could be an advantage in Fe‐limited environments, it may constitute a serious risk since they are gates for exogenous DNA and proteins. FhuA was first described to be essential for the infection of the phage T1 (T‐one A, named TonA) and it was later shown to be a TonB‐dependent protein involved in the transport of ferrichrome and colicin M, (Braun, 2009, and references therein). Other bacteriophages employ also TonB‐dependent gates (Rabsch *et al*., 2007).

In *E. coli, holo*‐Fur positively regulates the expression of iron storage and iron‐using proteins, such as bacterioferritin (Bfr) and superoxide dismutase (SodB), by repressing the expression of the small regulatory RNA RyhB (Massé and Gottesman, 2002). This does not seem to be the case in *P. pseudoalcaligenes*, since the expression of *sodB* (BN5_1139) and *bfr* (BN5_1112) diminished sevenfold in the *fur*− mutant. The absence of a Fur box upstream *sodB* and the universal stress protein family *uspA5* (BN5_1759) is consistent with the described positive regulation of *sodB* by *apo*‐Fur in *Helicobacter pylori* (Ernst *et al*., 2005). This hypothesis deserves further investigation since the absence of the proposed Fur box is not conclusive. The reduced expression of genes related to oxidative stress protection is consistent with the idea that, under iron limitation (simulated in the mutant), the oxidative stress should be lower. Obviously, the concentration of iron in the medium was normal and this can be the reason why the *fur*− mutant is especially sensitive to ROS (Becerra *et al*., 2014). A Fur box was observed in the upstream intergenic region of *bfr* and *bfd* (Fig. 4). This suggests a direct interaction with *holo*‐Fur. Bfr is an iron storage protein in bacteria previously identified as cytochrome b1 (Andrews *et al*., 1989), although it may also protect against oxidative stress (Carrondo, 2003). Bfd is a putative 2S‐2S ferredoxin protein. In *P. pseudoalcaligenes*, as in other bacteria, *bfd* and *bfr* genes are reciprocally regulated by iron availability via Fur. The expression of a protein responsible for the intracellular accumulation of iron (Bfr) decreases under iron‐limited conditions, while the expression of another protein responsible for its mobilization (Bfd) increases. In total, 17 proteins related to iron storage and metabolism and oxidative stress response were affected in the *fur*− mutant, six of them negatively (Table S2).

Proteins comprising the electron transport respiratory chain contain an important pool of iron as constituent of cytochromes and iron–sulfur centres. Therefore, the regulation of genes coding for the terminal oxidases in the *fur*− mutant (Table S2, Fig. 3) can also be discussed in the context of iron sparing strategies. The genome of *Pseudomonas pseudoalcaligenes* CECT 5344 carries out coding sequences for six terminal oxidases, three quinol oxidases and thee cytochrome oxidases (Luque‐Almagro *et al*., 2013). It also contains a truncated *cyoA* gene. The estimated number of iron atoms for the terminal oxidases Cyo, Cio, Aa3, Cbb3‐1 y Cbb3‐2, based on their composition (Thöny‐Meyer, 1997), is 2, 3, 3, 5 and 5 respectively. The downregulation of the *cio2* operon (Table 2) can be interpreted in this context. In *P. aeruginosa,* Fur regulates the expression of *cyo* in a similar way (Arai, 2011). Since *P. pseudoalcaligenes* CECT 5344 does not possess a functional Cyo, Cio2 may well substitute this function. Moreover, it is worth noting that the quinol oxidases, in comparison with the cytochrome oxidases, bypass the *bc1* complex and cytochrome *c*, thus providing an extra saving of iron. In *P. pseudoalcaligenes*, the cytochrome *cbb*_*3*_ oxidase is encoded, like in other Pseudomonadaceae (Pitcher and Watmough, 2004), by a tandem repetition of two *ccoNOQP* operons (Luque‐Almagro *et al*., 2013). The expression of the *cbb*_*3*_‐2 operon became downregulated in the *fur*− mutant (Table S2). A similar behaviour has been described for the homologous operon in *P. putida* under elevated O_2_ pressure (Follonier *et al*., 2013). In addition to the iron, many other environmental signals converge in regulating the expression of the terminal oxidases, such as copper and oxygen concentration, carbon and nitrogen sources, stationary phase or redox status (Arai, 2011). No Fur boxes were detected in the upstream intergenic sequences of these Fur‐regulated terminal oxidases.

By regulating the expression of regulatory genes, Fur expands its regulatory circuit and accentuates the hierarchy of Fur as master regulator. Since iron is an essential constituent of central biological processes, the connection of Fur with regulatory elements may allow a fine‐tune of the iron metabolism [(Miethke and Marahiel, 2007; Nies *et al*., 2007; Cornelis *et al*., 2009; Cornelis, 2010) and references therein]. Extracytoplasmic function (ECF) sigma factors are especially interesting because they respond to environmental signals without entering inside the cell. FiuA is responsible for the transport of the heterologous siderophore ferrichrome, and it is involved in a signalling pathway that regulates its own synthesis in response to the presence of the exogenously synthetized ferrichrome (Llamas *et al*., 2006; Hannauer *et al*., 2010). In *P. pseudoalcaligenes,* the expression of *fiuA* is induced by cyanide (Becerra *et al*., 2014). Figure 5 schematizes the putative regulatory and biochemical pathway involved in the transport of iron mediated by FiuIRA in *P. pseudoalcaligenes* CECT 5344. This figure is based on the results of both the present study and the bibliographic information. Under iron‐replete conditions, the system is supposed to be repressed by *holo*‐loaded Fur (Fig. 5A). The regulatory cascade began in the absence of iron, or the Fur protein itself (*fur*− mutant), allowing the expression of *fiuA* and *fiuIR* (Fig. 5B, Table S2). The presence of the signal siderophore in the extracellular medium, sensed by FiuA, arrests the anti‐sigma factor FiuR, thus allowing FiuI to promote the expression of its target genes (Fig. 5C). Obviously, these putative genes cannot be detected in the transcriptome but, since this bacterium does not produce siderophores, we can hypothesize which genes could be the target in addition to *fiuA* itself: the genes necessary for the transport across the inner membrane. This mechanism resembles a molecular relay, because it uses a low‐level control signal to switch a much higher circuit.

The genome of *P. pseudoalcaligenes* CECT 5344 harbours two CRISPR/Cas systems (Acera, 2011; Luque‐Almagro *et al*., 2013). It was the systematic exploration of prokaryotic genomes that permitted its discovery and role assignation as a genetic immune system to the CRISPR/Cas sequences (Mojica and Rodriguez‐Valera, 2016). The CRISP/Cas system can be considered as an acquired immunity system against exogenous genetic elements such as plasmidic DNA and viruses (Barrangou and Horvath, 2009; Horvath and Barrangou, 2010). The Cas proteins are located flanking the CRISPR sequences, and their function is processing the exogenous RNA or DNA (Hale *et al*., 2009). This system allows bacteria to adapt to the environment by Lamarckian inheritance, keeping track of genomic encounters. This probably helps to fine‐tune the delicate equilibrium between conservation (maintaining the status quo) and variation (providing novel genomic features and phenotypic properties in bacterial populations) (Mojica and Rodriguez‐Valera, 2016). Siderophores protect against colicins (Wayne *et al*., 1976), and both colicins and phages use receptors for iron uptake as entrance gates (Braun *et al*., 1976). These gates can be exploited to improve the antibiotic effectivity against pathogenic resistant strains (Mislin and Schalk, 2014).

Horizontal gene transfer (HGT) is the principal driving force in early cellular evolution (Woese, 2002), but once a certain limit of metabolic fitness is reached, evolution probably becomes conservative (self‐replicating), avoiding innovative events though DNA exchange that could be invasive or even parasitic. CRISPR may play an important role in this scenario. Here, we show two molecular mechanisms that fine‐tune conservation and evolution. First, the limitation of iron, detected by the master regulator Fur, induces (de‐represses) the expression of siderophore receptors that can serve as gates for exogenous DNA. Second, limitation of essential nutrients, included iron, represses the expression of genes responsible for the degradation of foreign DNA, thus allowing successful HGT. The presence of cyanide triggers both mechanisms at the same time, and it is the condition that most represses the expression of *cse1* (Fig. 6). It is remarkable that cyanide could have been essential in the evolution of life in the prebiotic world (Oró and Kimball, 1961; Patel *et al*., 2015). Therefore, cyanide makes this bacterium lower its guard during a discrete time. If the event (HGT) fruitfully succeeds, the stress will disappear and the system will turned back to safe levels. This equilibrium seems to be an example of ‘The Darwinian Threshold’ (Woese, 2002) in the molecular scope.

## Experimental procedures

### RNA isolation and quality assignment

Both the wt of *P. pseudoalcaligenes* CECT 5344 R1 and the *fur*− mutant were grown at 30°C in LB medium from three independent starter cultures. At the mid‐exponential phase (O.D._600_ = 0.3), 10 ml of each sample was collected and harvested by centrifugation. Total RNA was isolated using the Aurum Total RNA Mini Kit (Bio‐Rad, Hercules, CA, USA), and the purified RNA was treated with ‘DNase Treatment and Removal’ reagents (Ambion, Foster City, CA, USA), according to the manufacturer's instructions. After DNase treatment, a MICROB*Express*™ Bacterial mRNA Enrichment Kit (Ambion) was used to remove bacterial rRNA from the total RNA samples.

RNA was quantified using a NanoDrop1000 spectrophotometer (Thermo Fisher Scientific, Waltham, MA, USA), and the purity and integrity of RNA samples was measured using an Agilent 2100 Bioanalyzer with the RNA 6000 Nano LabChip Kit (Agilent Technologies, Santa Clara, CA, USA). All samples displayed a 260/280 ratio > 2.0 and RNA integrity numbers ≥ 9.

### RNA sequencing

RNA‐sequencing libraries were generated by Sistemas Genómicos (Valencia, Spain). mRNA samples were used to generate whole transcriptome libraries for sequencing on the SOLiDv4 platform, following the manufacturer's recommendation (Life Technologies, Carlsbad, CA, USA). No RNA‐spike in controls was used. Amplified cDNA quality was analysed by the Bioanalyzer 2100 DNA 1000 Kit (Agilent Technologies) and quantified using the Qubit 2.0 Fluorometer (Invitrogen, Carlsbad, CA, USA). The whole transcriptome libraries were used for making SOLiD templated beads following the SOLiD Templated Bead Preparation guide. Bead quality was estimated based on WFA (workflow analysis) parameters. Samples were sequenced using the 50625 paired‐end protocol, generating 50 nt + 35 nt (paired‐End) + 5 nt (barcode) sequences. Quality data were measured using software SETS parameters (SOLiD Experimental Tracking System, Life Technologies).

### Computational analysis of RNA‐seq data

The initial whole transcriptome paired‐end reads obtained from sequencing were mapped against the latest version of the *P. pseudoalcaligenes* CECT 5344 (Wibberg *et al*., 2014) using the Life Technologies mapping algorithm (http://www.lifetechnologies.com/), version 1.3. [http://solidsoftwaretools.com] in paired ends and whole transcriptome analysis. Bad quality reads (Phred score, 10) were eliminated using PicardTools software, [http://picard.sourceforge.net] (McKenna *et al*., 2010). Subsequently, isoforms and gene prediction were estimated using the cufflinks method (Trapnell *et al*., 2010) and the expression levels were calculated using the htseq software, version 0.5.4p3 (Anders and Huber, 2010). This method eliminates the multimapped reads, and only the unique reads are considered for gene expression estimation. Edge method, version 3.2.4, was applied for differential expression analysis between conditions (Robinson and Oshlack, 2010). Transcripts with *P*‐values < 0.05 and with fold change < −4 or > 4 were considered differentially expressed between mutant and wild‐type strains.

Transcripts were annotated. *P. pseudoalcaligenes* CECT 5344 R1 was selected in the protein database UniProt [http://www.uniprot.org/]. Each transcript was associated with the keywords from the database. To identify possible non‐coding RNAs, unknown transcripts were annotated by sequence homology against UniProt database and Rfam database [http://rfam.sanger.ac.uk/].

The complete genome of *P. pseudoalcaligenes* CECT5344 (Wibberg *et al*., 2014) was used as reference although many genes were manually annotated using GenDB 2.4 (Meyer *et al*., 2003). The genomics tool EDGAR was applied to comparative analysis and synteny analysis (Blom *et al*., 2009).

*Pseudomonas* genome database (Winsor *et al*., 2016) was also routinely used.

### Motif identification

Putative Fur‐DNA binding motifs were generated using MEME (version 5.0.4). Input sequences were derived from manually observed regions in the 200 bp upstream the Fur‐regulated genes.

## Conflict of interest

None declared.

## Supporting information


**Table S1**. Full transcriptome of the fur‐ mutant of *P. pseudoalcaligenes* CECT 5344. The genes were ordered first according to the accuracy of the value (from lowest to highest *P*‐val), and from de highest to the lowest FoldChange in a second level.

**Table S2**. Reduced transcriptome of the fur‐ mutant of *P. pseudoalcaligenes* CECT 5344. The genes from table S2 with a (*P*‐value < 0.05) were grouped in operons and assigned to the functional categories indicated in the table. For details, see the text.
